# Treg and immune proliferative programs characterize anti-PD-1-responsive endometrial cancer: an integrative single-cell, spatial transcriptomic, and TCGA-UCEC reanalysis

**DOI:** 10.3389/fgene.2026.1942724

**Published:** 2026-10-22

**Authors:** Yuan Zheng, Jingsheng Chen, Qing Wang, Qianqian Li

**Affiliations:** 1 Department of Gynecology, Yiwu Central Hospital, Yiwu, Zhejiang, China; 2 Department of Oncology, Ezhou Central Hospital, Ezhou, Hubei, China; 3 Department of Oncology, Haimen People’s Hospital, Nantong, Jiangsu, China

**Keywords:** anti-PD-1 therapy, endometrial cancer, immune microenvironment, microsatellite instability high, qRT-PCR, single-cell transcriptomics, spatial transcriptomics, TCGA-UCEC

## Abstract

**Background:**

The responses to programmed cell death protein 1 (PD-1)-based therapy in advanced endometrial cancer are heterogeneous. The cellular programs associated with such responses remain incompletely defined, particularly when the immune cell composition, spatial expression patterns, and population-level transcriptomic features are considered together.

**Methods:**

Publicly available TCGA-UCEC and GSE251923 data were reanalyzed using bulk transcriptomics, single-cell RNA sequencing (scRNA-seq), and the Visium expression space platform. The GSE251923 dataset included one anti-PD-1 responder and one non-responder; hence, all comparisons between these two cases are descriptive. Prespecified immune- and proliferation-related signatures were evaluated across the datasets, and the Visium clusters were assigned broad marker-based expression compartments. Exploratory quantitative reverse transcription polymerase chain reaction (qRT-PCR) assays were used to examine selected transcripts in the Ishikawa and HEC-1A endometrial cancer cell lines under 1% O_2_ hypoxia and in primary neutrophils exposed to endometrial-cancer ascites-conditioned medium. These assays were used as ancillary models of condition-associated transcription; neither hypoxia nor ascites exposure was treated as a direct model of clinical immune checkpoint inhibitor response.

**Results:**

After quality control, we obtained 16,725 single cells that were assigned to 14 populations. Compared with the non-responder sample, the responder sample contained higher proportions of CD8 T cells [+3.1 percentage points (pp)], regulatory T cells (Tregs; +3.3 pp), B cells (+6.7 pp), plasma cells (+5.4 pp), CD4 T cells (+5.9 pp), and proliferating cells (+7.7 pp); the non-responder sample dominantly contained epithelial/tumor and endothelial cells. In TCGA-UCEC (553 tumors and 35 normal tissues), a high Treg signature was associated with longer overall survival [log-rank *p* = 0.047; hazard ratio (HR) = 0.58; 95% confidence interval (CI): 0.34–1.00], whereas a high proliferation signature was associated with shorter overall survival (HR = 1.72; 95% CI: 1.01–2.94; *p* = 0.046). The responder Visium section showed descriptively higher Treg and proliferation scores with lower epithelial and extracellular matrix scores. Under hypoxia, the two endometrial cancer cell lines showed higher *MKI67*, *FOXP3*, *CXCL13*, *PDCD1*, and *CTLA4* transcript levels; the ascites-conditioned medium increased *PAD4* and *ELANE* expressions in primary neutrophils.

**Conclusion:**

The integrated analyses helped identify Treg- and proliferation-related patterns that differed between two anti-PD-1-treated endometrial cancers and were directionally contextualized in TCGA-UCEC. The qRT-PCR assays provide ancillary evidence of condition-associated transcriptional changes but do not validate immune cell states, clinical response mechanisms, or functional neutrophil extracellular trap formation. These findings are hypothesis-generating and require confirmation in independent treatment-annotated cohorts.

## 1 Introduction

Endometrial cancer is the most common gynecologic malignancy in developed countries, and its incidence has continued to increase alongside the prevalence of obesity and metabolic diseases (Sung et al., 2021; Morice et al., 2016). Although many patients with endometrial cancer are diagnosed at the early stage, advanced and recurrent disease remains difficult to treat and generally requires systemic therapy (Concin et al., 2021; Siegel et al., 2022). Programmed cell death protein 1 (PD-1)-based regimens have changed the treatment landscape, with pembrolizumab-containing strategies showing clinical benefits in advanced endometrial cancer (Eskander et al., 2023; Makker et al., 2022). As the use of such therapies continues to expand, the treatment selection must account for not only the response likelihood but also clinically important immune-related toxicities, including the uncommon but potentially severe complication of myocarditis (Salem et al., 2018; Moslehi et al., 2018; Johnson et al., 2016; Mahmood et al., 2018; Lyon et al., 2022).

The immune microenvironment of endometrial cancer is heterogeneous: the tumor-infiltrating lymphocytes, regulatory T cells (Tregs), myeloid cells, stromal elements, and vascular compartments may coexist in different proportions, and their functional states are not fully captured by a single biomarker. The response advantage observed in mismatch-repair-deficient or microsatellite-instability-high (MSI-H) disease suggests that the tumor genotype and immune context are closely linked; however, substantial variations remain within the molecular subgroups (Eskander et al., 2023; Makker et al., 2022). Macrophages, dendritic cells, and neutrophil-lineage populations can further shape T cell functions and tumor immune escape (Coffelt et al., 2016; Fridlender et al., 2009; Hedrick and Malanchi, 2022). In advanced peritoneal disease, malignant ascites may add another soluble and cellular compartment that could alter tumor–myeloid interactions (Kipps et al., 2013).

Neutrophil extracellular traps (NETs) have attracted attention as a possible link between inflammation, tumor progression, and impaired antitumor immunity. Tumor-derived chemokines can promote neutrophil recruitment and NET-associated responses, and the NET structures may reduce the access or activities of cytotoxic immune cells (Xiao et al., 2021; Teijeira et al., 2020). However, the question of whether ascites-associated neutrophil transcription in endometrial cancer is related to these processes remains uncertain and requires functional rather than transcript-only assessment.

Single-cell RNA sequencing (scRNA-seq) enables resolution of the immune and stromal populations at the cellular level while spatial transcriptomic approaches provide tissue-level expression context (Tang et al., 2009; Ståhl et al., 2016; Papalexi and Satija, 2018). Their integration has been used to examine cellular architectures and intercompartmental variations in several solid tumors (Wu et al., 2021; Moses and Pachter, 2022; Moncada et al., 2020; Izar et al., 2020). Bulk datasets, such as TCGA-UCEC, contain additional cohort-level information on the molecular subtype and survival. Therefore, cross-dataset analysis can help prioritize programs that recur across analytical scales, although the directional consistency alone cannot establish a common mechanism.

In this study, we reanalyzed scRNA-seq and Visium data from the GSE251923 dataset, which contains one endometrial cancer responder and one non-responder treated with anti-PD-1 therapy; we then placed the observed patterns in the context of TCGA-UCEC. We focused on Treg, cytotoxic T cell, myeloid, and proliferation-related programs, together with marker-based interpretation of the Visium clusters. Exploratory quantitative reverse transcription polymerase chain reaction (qRT-PCR) assays were performed as ancillary experiments to assess the transcriptional responses to hypoxia and ascites-conditioned exposures; these were not treated as direct models of clinical response or non-response.

## 2 Methods

### 2.1 Study design and data sources

The present study entails an exploratory reanalysis of two publicly available transcriptomic resources and ancillary qRT-PCR assays. The gene-level transcripts-per-million (TPM) expression data and clinical annotations for TCGA-UCEC were obtained from the Genomic Data Commons. The downloaded matrix contained 19,938 genes across 589 samples. After removing one recurrent tumor sample, we included 553 primary tumors and 35 solid-tissue normal samples. The MSI category information was available for 379 tumors: 127 MSI-H, 229 microsatellite-stable (MSS), 20 MSI-low, and 3 indeterminate. Binary MSI analyses were performed using the 356 tumors classified as MSI-H or MSS. The overall survival analyses included 377 tumors with non-missing survival information, among which 57 deaths were recorded.

The GSE251923 dataset was obtained from the Gene Expression Omnibus (GEO; accessed 10 August 2026) database. This dataset includes scRNA-seq and Visium data from two patients with endometrial cancer treated with anti-PD-1 therapy, where case A is a responder and case B is a non-responder. The samples analyzed here were GSM7990051 (responder scRNA-seq), GSM7990052 (non-responder scRNA-seq), GSM7990053 (responder Visium), and GSM7990054 (non-responder Visium). The source data contained 17,596 cells and 8,103 Visium spots. Because each response category was represented by one patient and one tissue section, the cell- and spot-level observations were not treated as independent patient-level replicates.

The TPM values from TCGA were confirmed to be on a linear scale and were transformed once as log_2_(TPM+1). The single-cell and Visium count matrices were then normalized using the normalize_total feature in scanpy toolkit, with a target sum of 10,000 followed by log1p transformation.

### 2.2 TCGA-UCEC bulk transcriptomic analysis

The tumor-versus-normal and MSI-H-versus-MSS differential expression analyses were performed using the Mann–Whitney U test on log_2_-transformed TPM values. Then, the Benjamini–Hochberg correction was applied, and genes meeting a false discovery rate (FDR) < 0.05 and an absolute log_2_ fold change (FC) ≥0.5 were considered significant. Finally, enrichment analysis was performed with the Enrichr tool using the KEGG_2021_Human and GO_Biological_Process_2021 libraries.

Seven prespecified immune/proliferation signatures were calculated as the arithmetic means of the normalized expression values of their member genes. The CD8 cytotoxic signature comprised *IFNG*, *GZMK*, *CD8B*, *CXCL13*, *CD8A*, *PRF1*, *GZMB*, *GNLY*, *GZMA*, and *NKG7*. The proliferation signature comprised *MKI67*, *TOP2A*, *PCNA*, *CENPF*, *UBE2C*, and *MCM2*. The remaining panels represented Treg, T cell exhaustion, myeloid-derived suppressor cell (MDSC), NET-neutrophil, and dendritic cell maturation programs and were calculated using the corresponding gene sets implemented in the original analysis workflow. The signature relationships were evaluated using the Spearman correlation value. The overall survival was examined using Kaplan–Meier curves after median dichotomization, log-rank tests, and univariable Cox proportional-hazards models implemented in the lifelines 0.30.3 library.

### 2.3 scRNA-seq analysis

The cells were retained when they contained 200–5,500 detected genes, a mitochondrial transcript fraction <20%, and at least 400 total counts. After quality control, we obtained 16,725 cells (13,390 from the responder and 3,335 from the non-responder), of which 16,029 genes remained. The workflow included a selection of 2,500 highly variable genes, principal component analysis (PCA) with 50 components, neighborhood graph construction with 15 neighbors and 30 principal components, uniform manifold approximation and projection (UMAP), and Leiden clustering at a resolution of 0.8. The cluster-versus-rest marker analysis was performed using the Wilcoxon rank-sum test. The cell identities were assigned from canonical lineage markers, followed by T cell subclustering at a resolution of 0.5 to yield 14 cell populations finally. The program scores were calculated as the mean of log1p-normalized expressions of the corresponding signature genes. The responder-versus-non-responder gene effects were summarized as the log_2_ ratios of the patient-mean log1p expressions. A mean expression threshold ≥0.05 and |log_2_FC| ≥0.5 was used as a descriptive screen. No cell-level *p*-value was interpreted as evidence of a patient-level response difference.

### 2.4 Visium expression space analysis and marker-based compartment annotation

The Visium spots were retained when they contained at least 200 detected genes, a mitochondrial transcript fraction <30%, and at least 500 total counts. Thus, all 8,103 spots passed the final workflow, including 4,204 from the responder and 3,899 from the non-responder sections. The normalized data were then analyzed using 2,000 highly variable genes, 40 principal components, a neighborhood graph with 12 neighbors and 20 principal components, UMAP, and Leiden clustering at a resolution of 0.8. The cluster interpretation was performed using the marker patterns displayed in Figure 1F: *EPCAM*, *KRT8*, and *PAX8* were used to identify epithelial/tumor-like expressions; *CD68* and *C1QA* indicated myeloid-associated expressions; *CD8A* and *FOXP3* indicated T-cell-associated signals; *MKI67* indicated proliferation; *COL1A1* indicated stromal/extracellular matrix (ECM) expression; *PECAM1* indicated endothelial expression; *MS4A1* and *MZB1* indicated B-cell-/plasma-cell-associated expressions. These labels describe the broad dominant components of the mixed Visium spots and were not treated as single-cell identities or formal deconvolution results. The signature scores were summarized by tissue section. Because the GEO dataset did not contain tissue position files, we used expression space UMAP projections in the analyses rather than anatomical tissue coordinates, and no claims of histologic colocalization were made.

**FIGURE 1:**
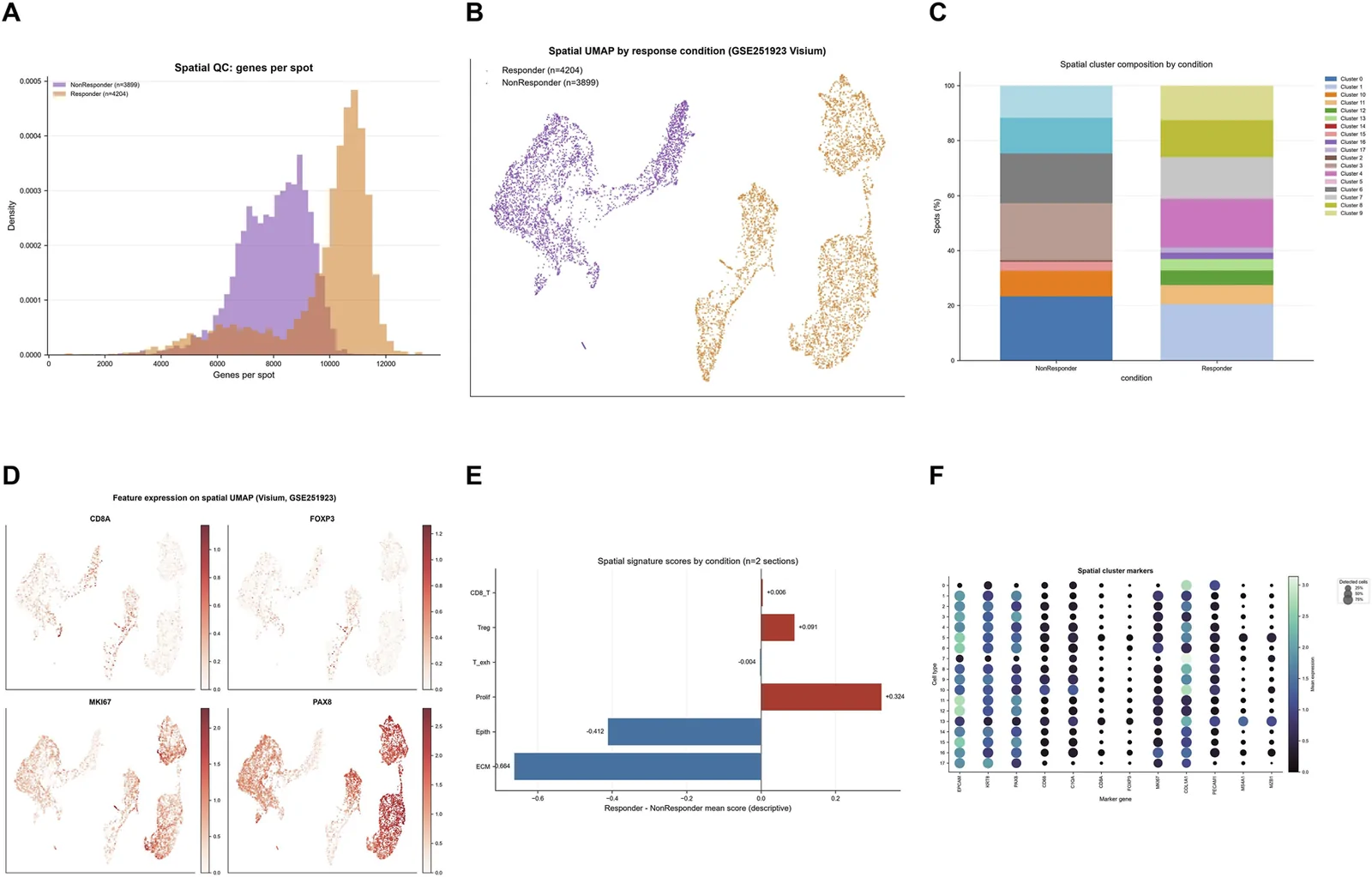
Exploratory GSE251923 Visium expression space analysis: **(A)** density distributions of the genes detected per Visium spot in the responder (n = 4,204) and non-responder (n = 3,899) sections; **(B)** expression space uniform manifold approximation and projection (UMAP) colored by response condition; **(C)** relative composition of the 18 Leiden clusters in the two sections; **(D)** *CD8A*, *FOXP3*, *MKI67*, and *PAX8* expressions in the expression space UMAP; **(E)** descriptive section-level signature differences calculated as responder-minus-non-responder levels for CD8 T cell, Treg, T cell exhaustion, proliferation, epithelial, and extracellular matrix (ECM) programs; **(F)** marker bubble matrix for clusters 0–17 using *EPCAM*, *KRT8*, *PAX8*, *CD68*, *C1QA*, *CD8A*, *FOXP3*, *MKI67*, *COL1A1*, *PECAM1*, *MS4A1*, and *MZB1*. The marker patterns support a coarse interpretation of most clusters as epithelial/tumor-like, clusters 0 and 7 as stromal-vascular-rich, cluster 10 as mixed epithelial-stromal/myeloid, cluster 13 as B/plasma-cell-enriched, and cluster 16 as proliferative epithelial-like. These labels represent putative dominant components of the mixed spots and not formal single-cell annotations. Because the GEO data did not contain tissue position files, panels **(B)** and **(D)** show expression space projections rather than anatomical tissue coordinates.

### 2.5 Cross-dataset contextualization

The effect-direction comparison was made using the 15,842 genes shared by the scRNA-seq and TCGA analyses. The overlap analysis compared scRNA-seq descriptive-effect genes (mean expression ≥0.05 and |log_2_FC| ≥0.5) with TCGA MSI-H-versus-MSS genes meeting FDR <0.05. The FC values were used to compare direction rather than absolute magnitude because the datasets differed in their expression scales, biological contrast, and statistical units. The signature distributions were standardized within each dataset before visualization. The MSI-H status was used as a molecular context and was not treated as a substitute for clinical anti-PD-1 response.

### 2.6 Exploratory qRT-PCR assays

Exploratory qRT-PCR assays were performed using the QuantStudio 3 system and TB Green Premix Ex Taq II. We studied the Ishikawa and HEC-1A endometrial cancer cell lines under normoxia and 1% O_2_ hypoxia, with *MKI67*, *FOXP3*, *CXCL13*, *PDCD1*, and *CTLA4* being the targets for measurement. The purpose of this experiment was to examine tumor cell transcriptional responses to hypoxic stress; hypoxia was not considered a model of anti-PD-1 response or non-response. The cell-line identities were verified by short tandem repeat profiling.

Primary neutrophils from three independent donors were exposed to endometrial-cancer ascites-conditioned medium or a corresponding control medium, and the *PAD4* and *ELANE* transcript levels were measured. This assay was aimed at assessing a peritoneal-disease-related neutrophil transcriptional response and not modeling immune checkpoint inhibitor (ICI) efficacy. The data were summarized as mean ± standard deviation, and comparisons were performed using unpaired t-tests with a two-sided *p*-value < 0.05. Because we did not assess the extracellular DNA, citrullinated histone H3, MPO-DNA complexes, microscopy, and rescue experiments, the *PAD4* and *ELANE* mRNA changes were interpreted as NET-associated transcriptions rather than functional evidence of NET formation.

### 2.7 Statistical analysis

Continuous distributions were compared using the Mann–Whitney U test unless specified otherwise. Multiple-testing corrections were performed using the Benjamini–Hochberg method. The overall survival analysis was performed using the log-rank test and univariable Cox proportional-hazards models. The qRT-PCR comparisons shown in Figure 2 were based on unpaired two-sided t-tests with *p* < 0.05 being considered statistically significant. For the GSE251923 dataset, patient or tissue section was treated as the biological unit; the cell and spot counts were not used to define independent replicates, and the response-related comparisons were interpreted descriptively.

**FIGURE 2:**
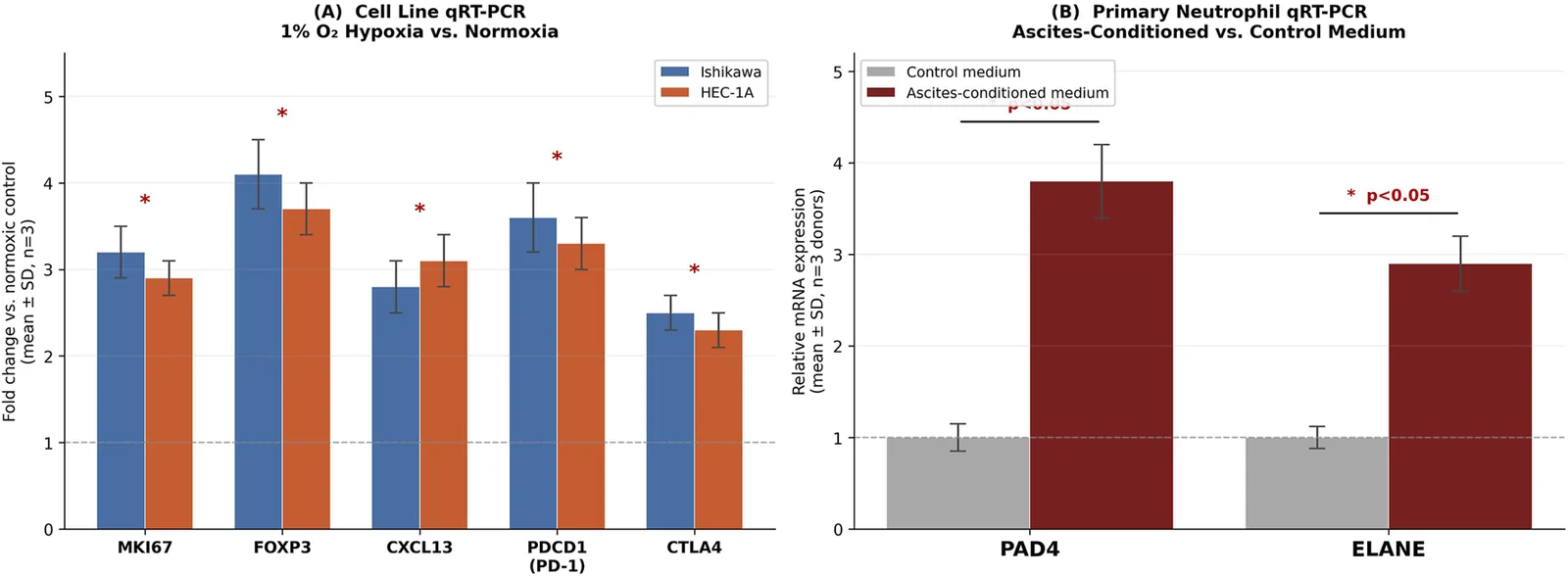
Exploratory qRT-PCR analysis of selected transcripts. **(A)** Fold-change expressions of *MKI67*, *FOXP3*, *CXCL13*, *PDCD1*, and *CTLA4* in the Ishikawa and HEC-1A endometrial cancer cell lines under 1% O_2_ hypoxia relative to normoxic controls. The data are presented as the mean ± SD from three independent experiments; the asterisks denote *p* < 0.05 as displayed. The assay evaluates tumor cell transcriptional responses to hypoxia and does not validate immune cell identity or anti-PD-1 response status. **(B)** Relative *PAD4* and *ELANE* mRNA expressions in primary neutrophils exposed to endometrial-cancer ascites-conditioned medium or control medium. The data are presented as the mean ± SD from three independent donors; *PAD4*, *p* = 0.012; *ELANE*, *p* = 0.028. These mRNA changes indicate a neutrophil extracellular trap (NET)-associated transcriptional tendency but do not demonstrate functional NET formation.

## 3 Results

### 3.1 TCGA-UCEC cohort structure and expression landscape

After excluding the single recurrent tumor sample, our TCGA-UCEC analysis included 553 primary tumors and 35 solid-tissue normal samples. The scale audit showed a median log_2_(TPM+1) value of 2.593, with a prominent low-expression peak and a broad non-zero-expression peak extending toward higher values, confirming that the linear TPM data had undergone a single log_2_(x+1) transformation (Figure 3A). PCA based on the 2,000 most variable genes could separate most tumors from normal samples, with PC1 and PC2 explaining 12.3% and 9.1% of the total variance, respectively (Figure 3B). The analyzed cohort comprised 588 samples in total, with an expected imbalance between the tumors and normal tissues (Figure 3C).

**FIGURE 3:**
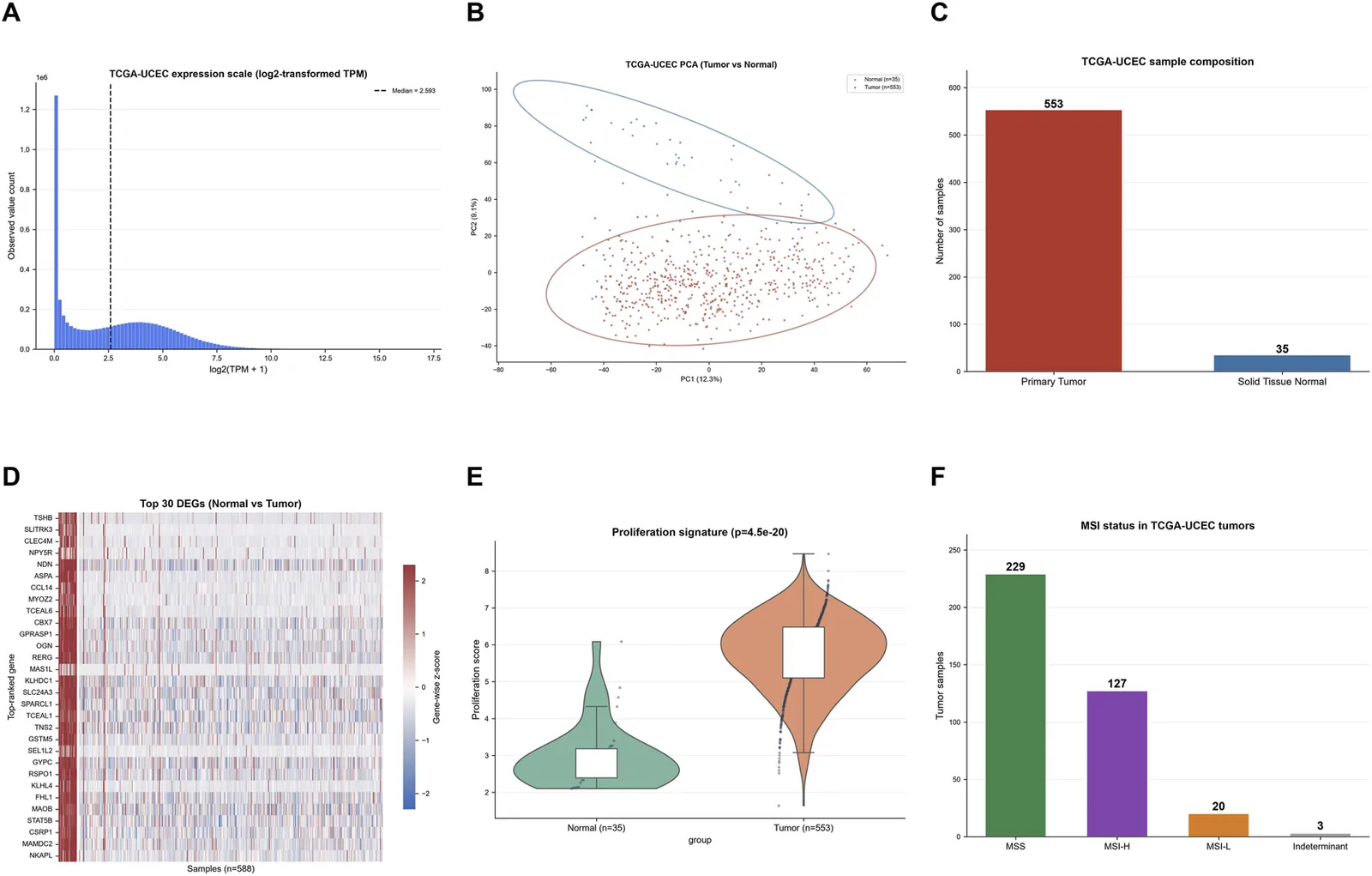
TCGA-UCEC cohort and expression landscape. **(A)** Distribution of log_2_(TPM+1) expression values after scale audit; the median transformed value was 2.593. **(B)** Principal component analysis of the 2,000 most variable genes. Each point represents one sample; the ellipses indicate 95% confidence regions. PC1 and PC2 explain 12.3% and 9.1% of the total variance, respectively. **(C)** Numbers of primary tumors (n = 553) and solid-tissue normal samples (n = 35). **(D)** Gene-wise z-score heatmap of the 30 top-ranked tumor-versus-normal differentially expressed genes, with normal samples ordered before tumors. **(E)** Proliferation signature, defined as the mean log_2_(TPM+1) value of *MKI67*, *TOP2A*, *PCNA*, *CENPF*, *UBE2C*, and *MCM2*, by tissue type (Mann–Whitney: *p* = 4.5e-20). **(F)** MSI-category counts among annotated tumors: MSI-H, n = 127; MSS, n = 229; MSI-L, n = 20; indeterminate, n = 3. The binary MSI-H-versus-MSS analyses involved 356 tumors with a definitive MSI-H or MSS status.

The heatmap of the top-30 differentially expressed genes (DEGs) showed a relatively uniform expression pattern for the normal tissues and greater heterogeneity among the tumors; most displayed genes, including *TSHB*, *SLITRK3*, *CCL14*, *OGN*, *SPARCL1*, *RERG*, and *FHL1*, had lower expression in the tumors (Figure 3D). The proliferation signature calculated using *MKI67*, *TOP2A*, *PCNA*, *CENPF*, *UBE2C*, and *MCM2* was markedly higher for the tumors than normal tissues (Mann–Whitney: *p* = 4.5e-20; Figure 3E). The MSI category information was available for 379 tumor samples, including 127 MSI-H, 229 MSS, 20 MSI-L, and 3 indeterminate samples; the binary MSI-H-versus-MSS analysis therefore included 356 tumors with a definitive MSI-H or MSS status (Figure 3F).

The tumor-versus-normal differential expression analysis resulted in 7,471 genes meeting the condition of FDR<0.05 and |log2FC| ≥0.5, of which 2,611 were upregulated and 4,860 were downregulated in the tumors (Figure 4A). The corresponding MA plot showed significant changes across a broad range of mean expression levels, with the largest FCs occurring mainly among genes with low-to-intermediate average expressions (Figure 4B). The genes with the largest positive effects included *MYBL2*, *SFN*, *PRAME*, *UBE2C*, *CDC20*, *BIRC5*, *TPX2*, *AURKB*, *TK1*, and *TOP2A*, whereas the most strongly downregulated genes were *CCL21*, *CHRDL1*, *RERG*, *RSPO1*, *PTGIS*, *OGN*, *CNN1*, *MYH11*, and *DES* (Figure 4C).

**FIGURE 4:**
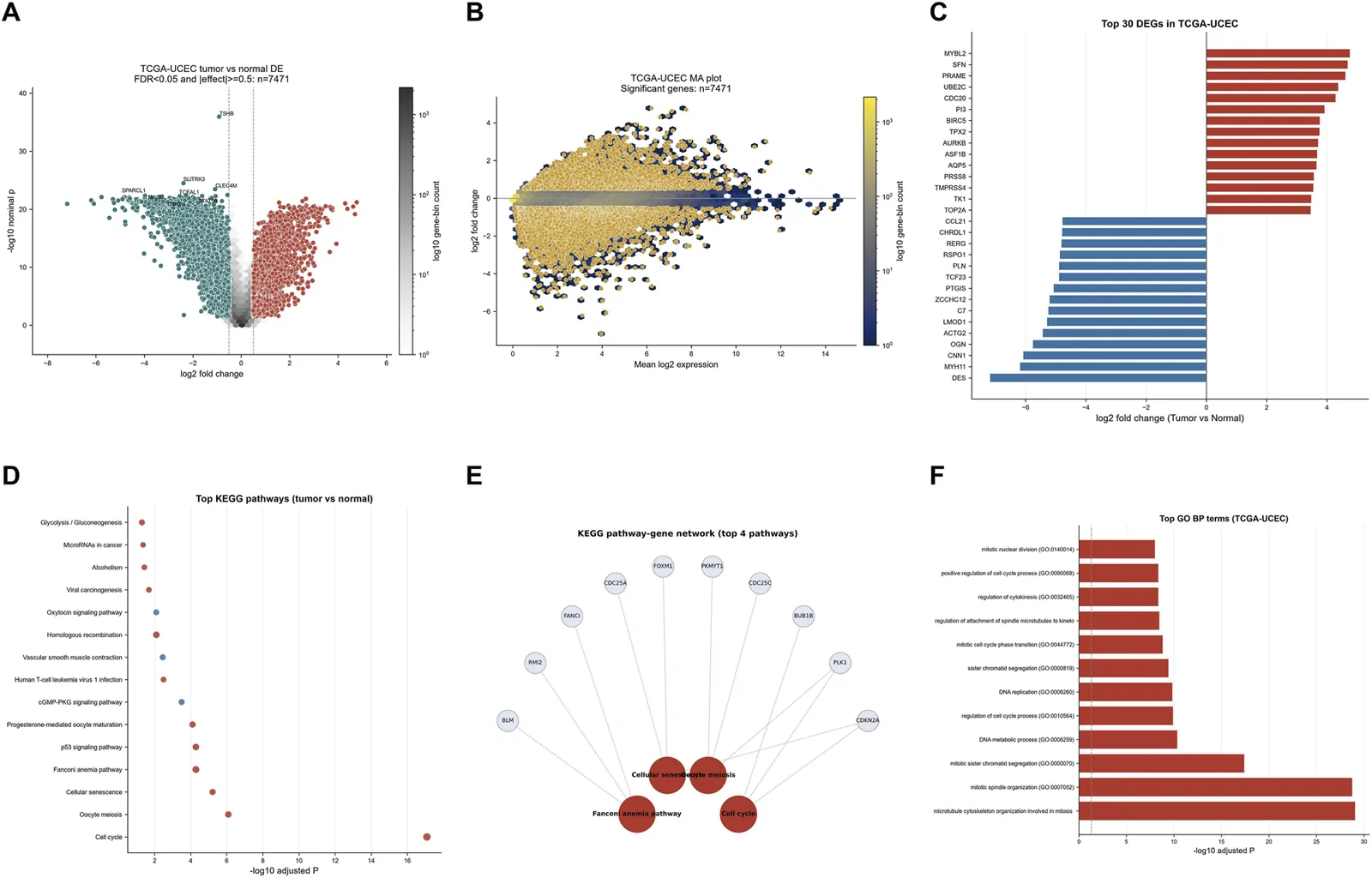
TCGA-UCEC tumor-versus-normal differential expression and enrichment. **(A)** Volcano plot of 19,938 genes. The x-axis shows log_2_FC(tumor minus normal), and the y-axis shows -log10 nominal *p*-value. The genes meeting FDR<0.05 and |log_2_FC| ≥0.5 are shown as significant (n = 7,471; 2,611 upregulated and 4,860 downregulated). **(B)** MA-style plot of log_2_FC against average log_2_ expression. **(C)** Top-30 genes with the largest absolute log_2_FC values, comprising 15 tumor-upregulated and 15 tumor-downregulated genes. **(D)** Leading KEGG pathways ranked by adjusted *p*-value. **(E)** Pathway-gene network connecting the four leading KEGG terms with shared contributing genes. **(F)** Leading GO biological process terms ranked by adjusted *p*-value.

KEGG enrichment of the tumor-associated DEGs was dominated by cell-cycle, oocyte meiosis, cellular senescence, Fanconi anemia, p53 signaling, homologous recombination, and related DNA replication or repair pathways (Figure 4D). The pathway-gene network showed extensive sharing of the mitotic checkpoint and DNA repair genes, including *BLM*, *RMI2*, *FANCI*, *CDC25A*, *FOXM1*, *PKMYT1*, *CDC25C*, *BUB1B*, *PLK1*, and *CDKN2A*, across the leading KEGG terms (Figure 4E). The GO biological process enrichment analysis further highlighted mitotic microtubule organization, spindle organization, sister chromatid segregation, DNA replication, cell-cycle transition, cytokinesis, and mitotic nuclear division as the prominent categories (Figure 4F). Together, these analyses show a prominent proliferative and mitotic transcriptional program in endometrial tumors.

### 3.2 Immune signatures, MSI status, and overall survival in TCGA-UCEC

The CD8 T cell signature displayed a broader distribution and more pronounced high-score tail in tumors than normal tissues, although the two distributions overlapped substantially (Figure 5A). Across the tumors, the CD8 T cell, Treg, and T cell exhaustion signatures were correlated strongly: the Spearman rho value was 0.90 for CD8 T cell vs. T cell exhaustion, 0.80 for CD8 T cell vs. Treg, and 0.87 for Treg vs. T cell exhaustion (Figure 5B). A separate myeloid-related axis was evident from the high correlation between the MDSC and NET-neutrophil signatures (rho = 0.88). In contrast, the proliferation signature showed little correlation with the immune signatures, with rho values close to zero.

**FIGURE 5:**
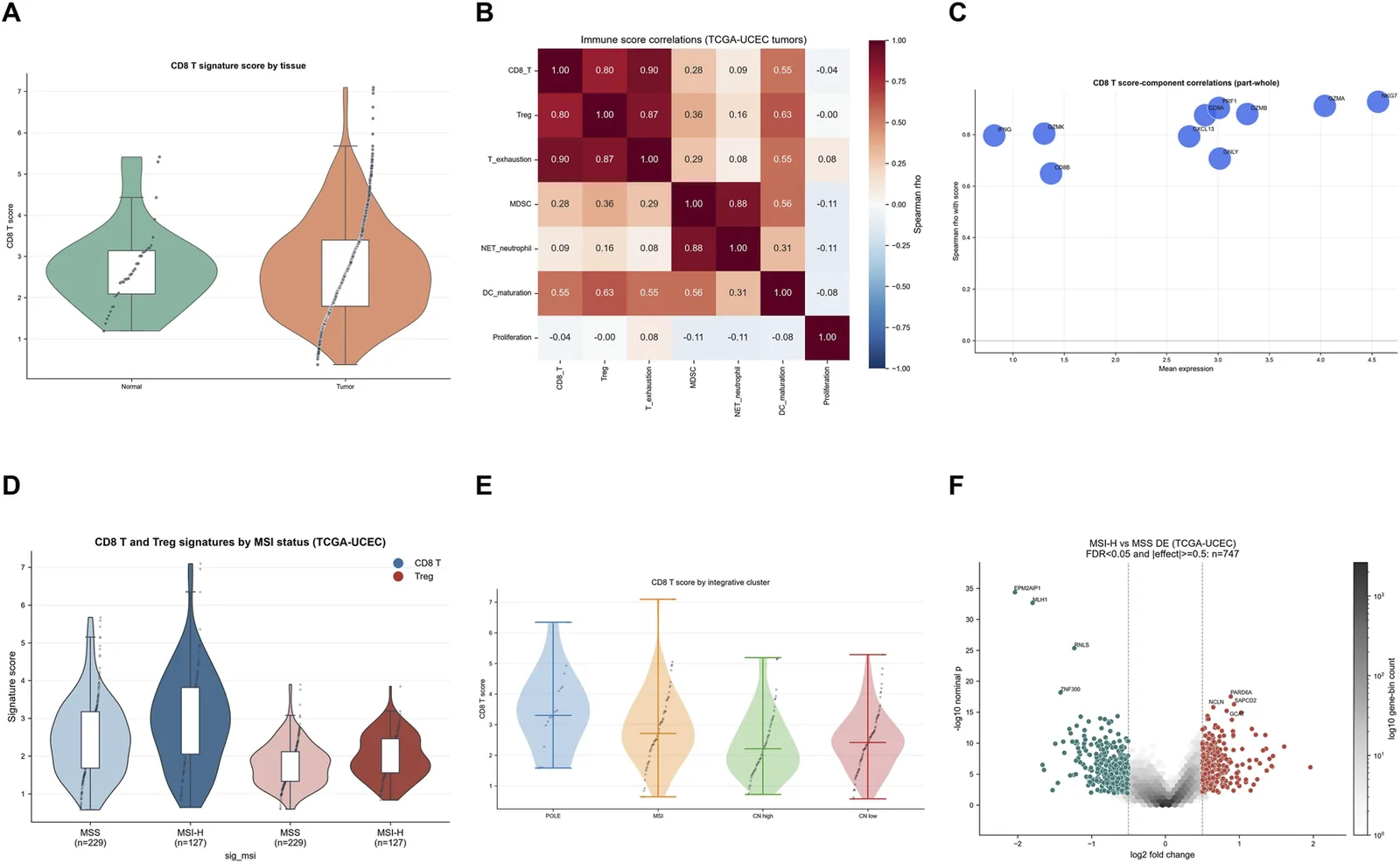
TCGA-UCEC immune signatures and MSI-related patterns. **(A)** CD8 T cell signature distributions in normal tissues (n = 35) and tumors (n = 553). **(B)** Spearman correlation matrix of the seven immune/proliferation signatures across tumors. **(C)** Correlations between the composite CD8 T cell score and its component genes; these part–whole correlations assess internal score consistency. **(D)** CD8 T cell and Treg signature distributions in MSS (n = 229) and MSI-H (n = 127) tumors. No T cell exhaustion score is displayed in this panel. **(E)** CD8 T cell scores across the POLE, MSI, CN-low, and CN-high integrative molecular clusters. **(F)** MSI-H-versus-MSS differential expression volcano plot. A total of 747 genes met FDR<0.05 and |log_2_FC| ≥0.5, including 328 upregulated and 419 downregulated genes in the MSI-H tumors.

All component genes of the CD8 T cell score were positively correlated with the composite score (Figure 5C): *NKG7* and *GZMA* showed the strongest correlations, followed by *PRF1*, *CD8A*, *GZMB*, *GZMK*, *IFNG*, *CXCL13*, *GNLY*, and *CD8B*. Because the composite score was calculated from these genes, such part–whole correlations were interpreted as an internal consistency check rather than an independent validation. The MSI-H tumors showed higher distributions of both CD8 T cell and Treg scores than the MSS tumors, although considerable within-group variation and overlap remained (Figure 5D). The CD8 T cell score was highest in the POLE integrative molecular cluster, followed by the MSI, CN-low, and CN-high tumors (Figure 5E). Using the combined threshold of FDR <0.05 and |log_2_FC| ≥0.5, the MSI-H-versus-MSS comparison showed 747 DEGs, including 328 upregulated and 419 downregulated genes in the MSI-H tumors (Figure 5F). Here, *MLH1*, *EPM2AIP1*, *RNLS*, and *ZNF300* were among the labeled negative-effect genes, whereas *PARD6A*, *NCLN*, *SAPCD2*, and *GCAT* were among the labeled positive-effect genes.

The overall survival data were available for 377 tumor cases, which included 57 deaths. The high CD8 T cell group showed a non-significant tendency toward longer survival than the low-score group (log-rank *p* = 0.0818; Figure 6A). A high Treg score was associated with longer overall survival in the median-split analysis (log-rank *p* = 0.0472; Figure 6B), whereas T cell exhaustion was not associated with overall survival (*p* = 0.508; Figure 6C). In the univariable Cox analysis, the hazard ratios (HRs) for the high-versus-low groups were 0.63 (95% confidence interval (CI): 0.37–1.07) for CD8 T cells, 0.58 (95% CI: 0.34–1.00) for Tregs, 0.84 (95% CI: 0.50–1.41) for T cell exhaustion, 1.29 (95% CI: 0.76–2.17) for MDSCs, 1.29 (95% CI: 0.76–2.18) for NET-neutrophils, 0.90 (95% CI: 0.53–1.52) for dendritic cell maturation, and 1.72 (95% CI: 1.01–2.94) for proliferation (Figure 6D). The high-Treg group included 21 deaths among 188 patients (11.2%) compared to 36 deaths among 189 patients (19.0%) for the low-Treg group (Figure 6E). The CD8 T cell scores were slightly higher in FIGO stage I/II than in stage III/IV disease, but this difference was not statistically significant (*p* = 0.0662; Figure 6F).

**FIGURE 6:**
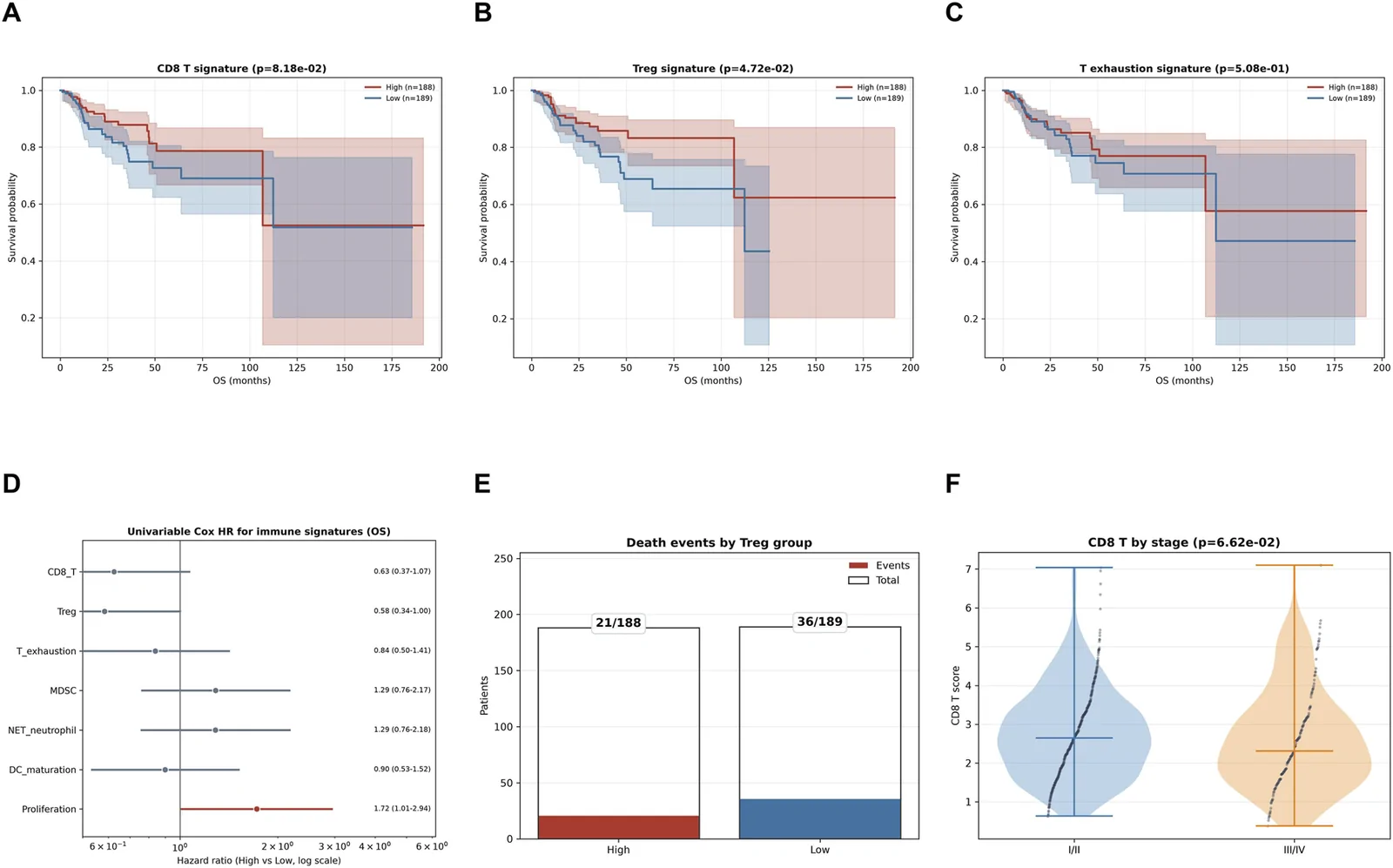
TCGA-UCEC overall survival analyses. **(A–C)** Kaplan–Meier overall survival curves after median dichotomization of the CD8 T cell (*p* = 0.0818), Treg (*p* = 0.0472), and T cell exhaustion (*p* = 0.508) signatures, respectively. The analysis included 377 tumors and 57 deaths. **(D)** Univariable Cox proportional-hazards estimates for the seven signatures, presented as hazard ratios with 95% confidence intervals. **(E)** Numbers of deaths and total patients in the high- and low- Treg groups. **(F)** CD8 T cell signature distributions in the FIGO stage I/II and stage III/IV tumors (*p* = 0.0662).

### 3.3 Single-cell landscape of the anti-PD-1-treated tumors

After quality control, a total of 16,725 cells were retained from the GSE251923 dataset, including 13,390 cells from the responder sample and 3,335 cells from the non-responder sample. The condition-colored UMAP shows marked separation between the two samples, indicating substantial differences in cellular composition and transcriptional structure; because each response category is represented by a single patient, this separation is not attributable to treatment response exclusively (Figure 7A). The cells were annotated into 14 populations as follows: epithelial/tumor cells (n = 7,644), myofibroblasts (n = 1,598), fibroblasts (n = 1,329), proliferating cells (n = 1,025), B cells (n = 894), plasma cells (n = 871), CD4 T cells (n = 834), endothelial cells (n = 676), macrophages/monocytes (n = 672), Tregs (n = 444), CD8 T cells (n = 425), mast cells (n = 162), proliferating T cells (n = 78), and lymphatic endothelial cells (n = 73) (Figure 7B).

**FIGURE 7:**
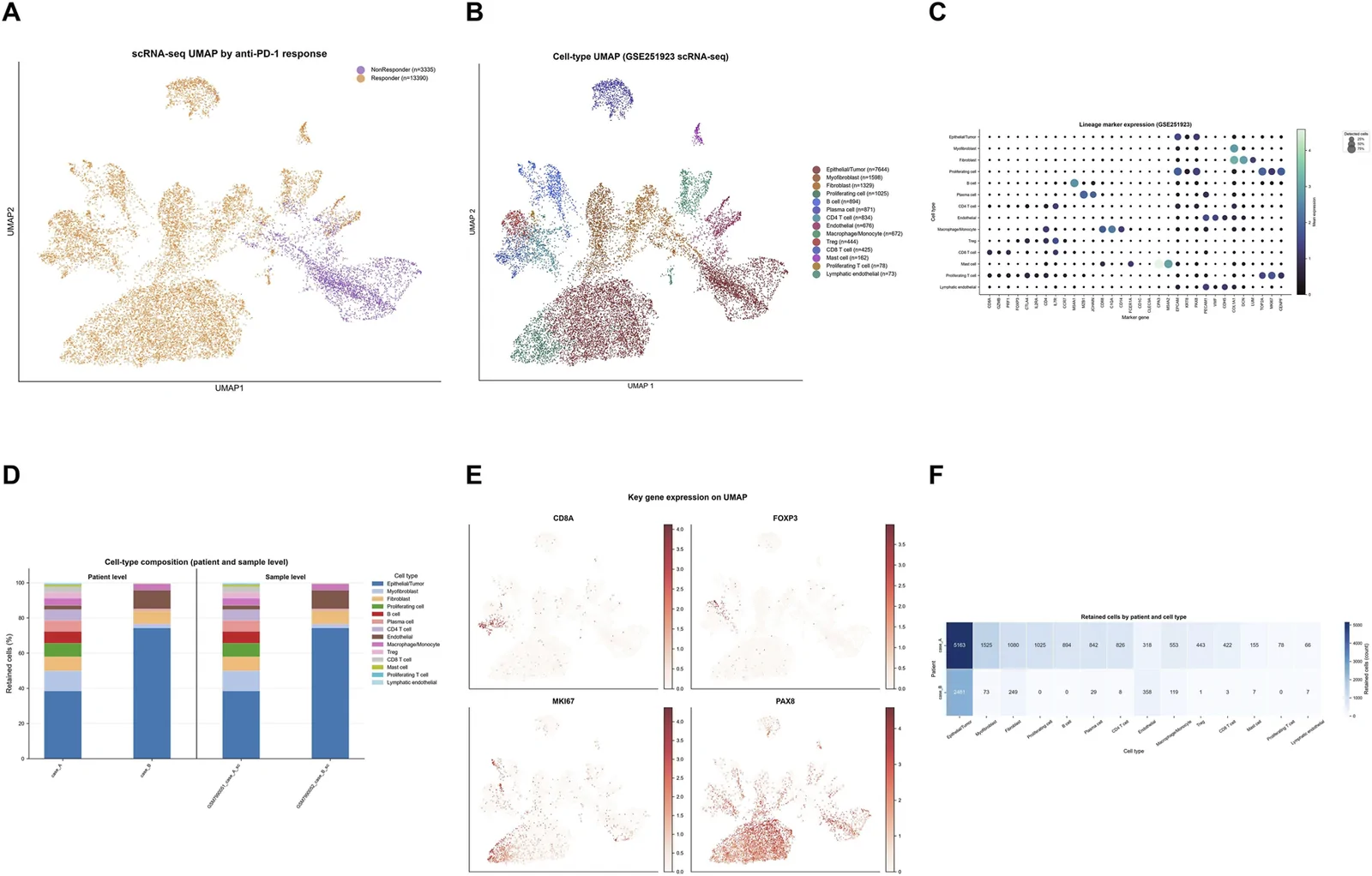
GSE251923 single-cell RNA-sequencing landscape and cell annotation. **(A)** UMAP colored by anti-PD-1 response condition: responder sample, n = 13,390 cells; non-responder sample, n = 3,335 cells. Because each condition contains data from only one patient, condition separation also captures patient-specific and technical differences. **(B)** UMAP colored by the 14 annotated cell populations. **(C)** Bubble matrix of representative lineage markers; the bubble size indicates the percentage of cells expressing the gene and color indicates mean expression. **(D)** Stacked proportions of the 14 cell populations at the patient/sample level. This panel shows the cell-type composition rather than marker-gene expression. **(E)** Feature UMAPs for *CD8A*, *FOXP3*, *MKI67*, and *PAX8.* **(F)** Retained-cell count for each annotated cell type in case A (responder) and case B (non-responder).

The marker-gene matrix supported the major lineage assignments (Figure 7C). The CD8 T cells expressed *CD8A*, *GZMB*, and *PRF1*; Tregs expressed *FOXP3*, *CTLA4*, and *IL2RA*; CD4 T cells expressed *CD4*, *IL7R*, and *CCR7*; B cells expressed *MS4A1*; plasma cells expressed *MZB1* and *JCHAIN*; macrophages/monocytes expressed *CD68*, *C1QA*, and *CD14*; epithelial/tumor cells expressed *EPCAM*, *KRT8*, and *PAX8*; endothelial cells expressed *PECAM1*, *VWF*, and *CDH5*; fibroblast-lineage cells expressed *COL1A1*, *DCN*, and *LUM*; and proliferating populations expressed *TOP2A*, *MKI67*, and *CENPF*. The stacked composition plot showed that the responder sample contained more diverse immune, stromal, and proliferative compartments, whereas the non-responder sample was dominated by epithelial/tumor and endothelial cells (Figure 7D). The feature UMAPs localized *CD8A* to the cytotoxic T cell region, *FOXP3* to the Treg region, *MKI67* to the proliferating cells and proliferating T cells, and *PAX8* to the epithelial/tumor compartment (Figure 7E). The patient-by-cell-type counts further demonstrated the marked imbalance between the two samples (Figure 7F). The responder sample contained 443 Tregs, 422 CD8 T cells, 894 B cells, 842 plasma cells, 826 CD4 T cells, 1,025 proliferating cells, and 78 proliferating T cells. The non-responder sample contained only 1 Treg and 3 CD8 T cells with no B cells, conventional proliferating cells, or proliferating T cells. Accordingly, the downstream comparisons involving these populations were treated as descriptive observations rather than patient-level statistical tests.

### 3.4 Descriptive single-cell comparison of the responder and non-responder samples

The cellular composition differences were most pronounced for the epithelial/tumor cells, which accounted for 38.56% of the responder and 74.39% of the non-responder samples, followed by endothelial cells, which accounted for 2.37% and 10.73%, respectively (Figure 8A). The responder sample had higher proportions of myofibroblasts (11.39% vs. 2.19%), proliferating cells (7.65% vs. 0%), B cells (6.68% vs. 0%), plasma cells (6.29% vs. 0.87%), CD4 T cells (6.17% vs. 0.24%), Tregs (3.31% vs. 0.03%), and CD8 T cells (3.15% vs. 0.09%). These percentage-point differences describe the two profiled tumors and do not constitute group-level estimates.

**FIGURE 8:**
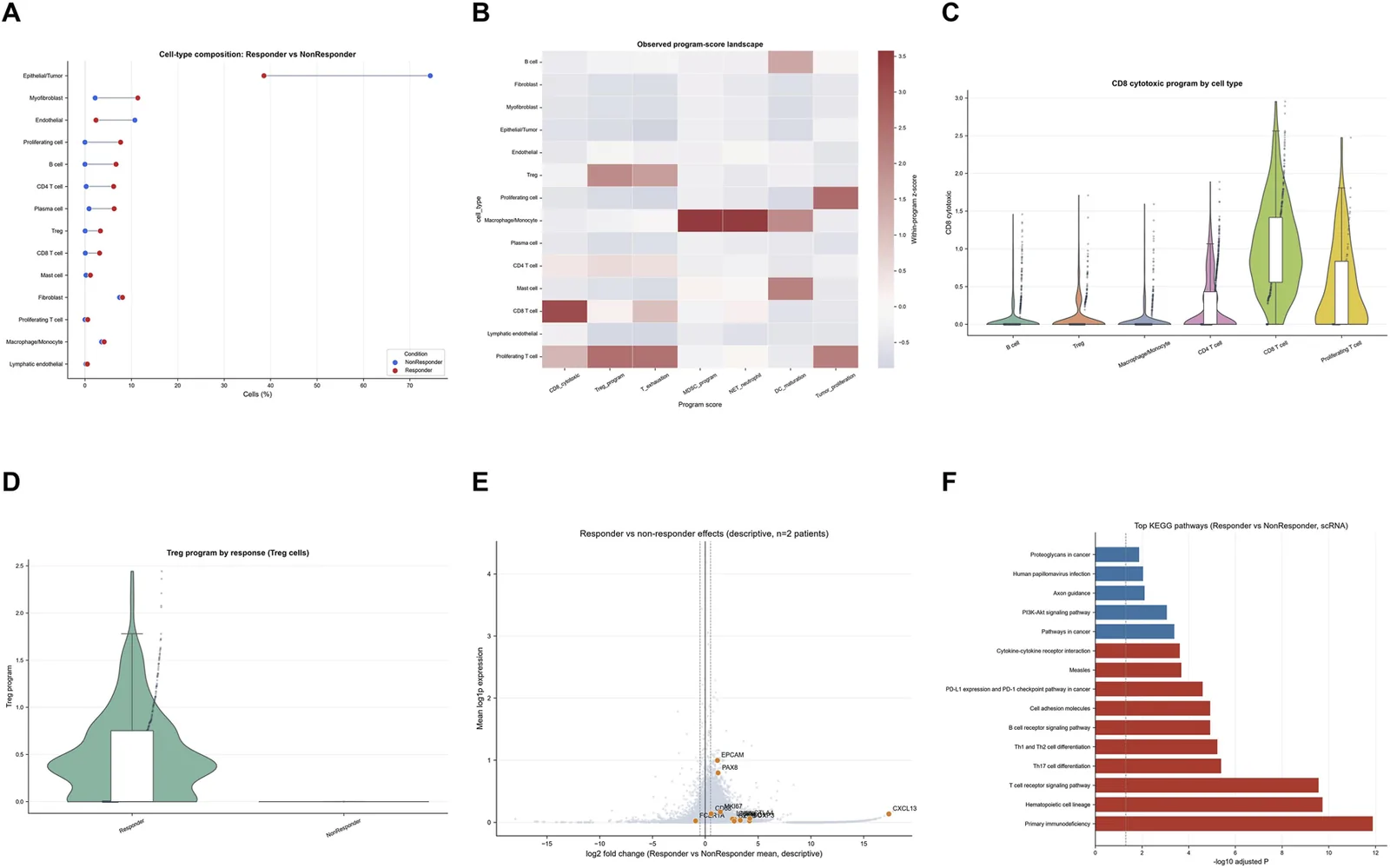
Descriptive single-cell comparison of the responder and non-responder samples. **(A)** Dumbbell plot of cell-type proportions in the responder and non-responder samples. The values describe two individual tumors and are not group-level estimates. **(B)** Cell-type-level z-scored program matrix for CD8 cytotoxicity, Treg, T cell exhaustion, MDSC, NET-neutrophil, dendritic cell maturation, and tumor proliferation programs. **(C)** CD8 cytotoxic program scores across selected cell populations. **(D)** Treg program score distribution within annotated Treg cells. The responder sample contributed 443 Tregs, whereas the non-responder sample contributed one Treg; this panel is descriptive and does not support patient-level inference. **(E)** Descriptive gene-effect plot showing log_2_ ratios of patient-mean log1p expression for responder vs. non-responder cells. The vertical lines indicate |log2FC| = 0.5. No patient-level significance testing was performed. **(F)** Leading KEGG terms enriched among the responder-associated descriptive-effect genes.

The program score patterns were broadly consistent with the annotated cell identities (Figure 8B). The CD8 cytotoxic program was highest in the CD8 T cells and was also high in the proliferating T cells; the Treg and T cell exhaustion programs were enriched in the Tregs and proliferating T cells; the MDSC and NET-neutrophil programs were strongest in the macrophages/monocytes; the tumor proliferation program was highest in the proliferating cells and proliferating T cells. Direct visualization of the CD8 cytotoxic score confirmed that the highest values occurred in the CD8 T cells, with a smaller high-score subset among the proliferating T cells (Figure 8C). Within the annotated Treg compartment, the responder sample contained 443 cells with a continuous range of Treg program scores, whereas the non-responder sample contributed a single Treg cell with a near-zero score (Figure 8D). This panel therefore establishes the presence of a Treg-program-positive population in the responder sample but does not support a reliable between-patient distributional comparison. The descriptive gene-effect plot showed higher patient-mean expressions of *CXCL13*, *CTLA4*, *FOXP3*, *PDCD1*, *CD8A*, and *MKI67* in the responder sample, whereas *FCER1A* showed a negative effect (Figure 8E). The very large effect estimates for *CXCL13* and other low-abundance genes should be interpreted cautiously because the near-zero expression in the non-responder sample can inflate the log2 ratio.

KEGG analysis of the responder-associated descriptive-effect genes highlighted primary immunodeficiency, hematopoietic cell lineage, T cell receptor signaling, Th17 differentiation, Th1/Th2 differentiation, B cell receptor signaling, cell-adhesion molecules, cytokine–cytokine receptor interaction, PD-L1 expression, and PD-1 checkpoint pathway in cancer (Figure 8F). These enrichments were directionally consistent with the greater lymphocyte representation in the responder sample, but the input effects were derived from only two patients.

### 3.5 Spatial transcriptomic characterization

After quality control, we retained 8,103 Visium spots including 4,204 from the responder and 3,899 from the non-responder sections. The responder section showed a right-shifted genes-per-spot distribution, with a major peak at approximately 10,500–11,000 detected genes per spot, whereas the non-responder section was concentrated mainly at approximately 7,000–9,500 genes per spot (Figure 1A). This systematic difference in transcript complexity was considered when interpreting the downstream section-level score differences. On the expression space UMAP, spots from the two sections formed almost completely separate groups (Figure 1B), reflecting the combined biological, compositional, technical, and patient-specific differences. The 18 Leiden clusters were also largely section-specific, with little sharing of the dominant clusters between the responder and non-responder sections (Figure 1C). The *CD8A* and *FOXP3* expressions were sparse, whereas *MKI67* expression was more prominent in several responder-associated UMAP regions and *PAX8* was broadly expressed across epithelial-associated regions in both sections (Figure 1D). Because the tissue position files were unavailable, these displays represent expression space UMAP projections and cannot be interpreted as anatomical colocalization, exclusion, or neighborhood relationships.

The section-level score differences, calculated as responder minus non-responder scores, were +0.006 for the CD8 T cells, +0.091 for the Tregs, −0.004 for T cell exhaustion, +0.324 for proliferation, −0.412 for the epithelial program, and −0.664 for the ECM program (Figure 1E). The largest positive difference was observed for proliferation, whereas the largest negative differences were observed for the ECM and epithelial programs; the CD8 T cell and T cell exhaustion scores were nearly unchanged. The spatial-cluster marker matrix enabled a coarse interpretation of the dominant transcriptional components without implying pure cell identities (Figure 1F). Most of the clusters expressed *EPCAM*, *KRT8*, and/or *PAX8* and were therefore considered epithelial/tumor-like. Clusters 0 and 7 showed strong *COL1A1* together with *PECAM1* and relatively low epithelial-marker expressions, consistent with stromal-vascular-rich spots. Cluster 10 showed *CD68*, *C1QA*, and *COL1A1* in addition to the epithelial markers, indicating a mixed epithelial-stromal/myeloid component. Cluster 13 showed the strongest *MS4A1* and *MZB1* signals and was interpreted as B/plasma-cell-enriched, whereas cluster 16 showed a relatively strong *MKI67* signal within an epithelial background and was interpreted as proliferative epithelial-like. *CD8A* and *FOXP3* expressions remained low and sparse across the clusters, and no discrete CD8-T-cell- or Treg-dominant spatial cluster was assigned. These labels represent putative dominant components of the mixed Visium spots rather than formal single-cell annotations or deconvolution results.

### 3.6 Cross-dataset contextualization

Across the 15,842 shared genes, the descriptive scRNA-seq responder-versus-non-responder effects showed only modest overall concordance with TCGA tumor-versus-normal effects, with the highest density of genes centered near zero in both dimensions (Figure 9A). When the selected immune and proliferation genes were compared between the scRNA-seq response contrast and TCGA MSI-H-versus-MSS contrast, we noted that *CXCL13*, *CD8A*, *GZMB*, *PRF1*, *FOXP3*, *CTLA4*, *IL2RA*, *PDCD1*, *LAG3*, *TIGIT*, *MKI67*, and *TOP2A* showed positive effects in both contexts, whereas *FCER1A* showed negative effects in both contexts (Figure 9B); *S100A8*, *S100A9*, and *CD33* showed discordant directions between the two comparisons.

**FIGURE 9:**
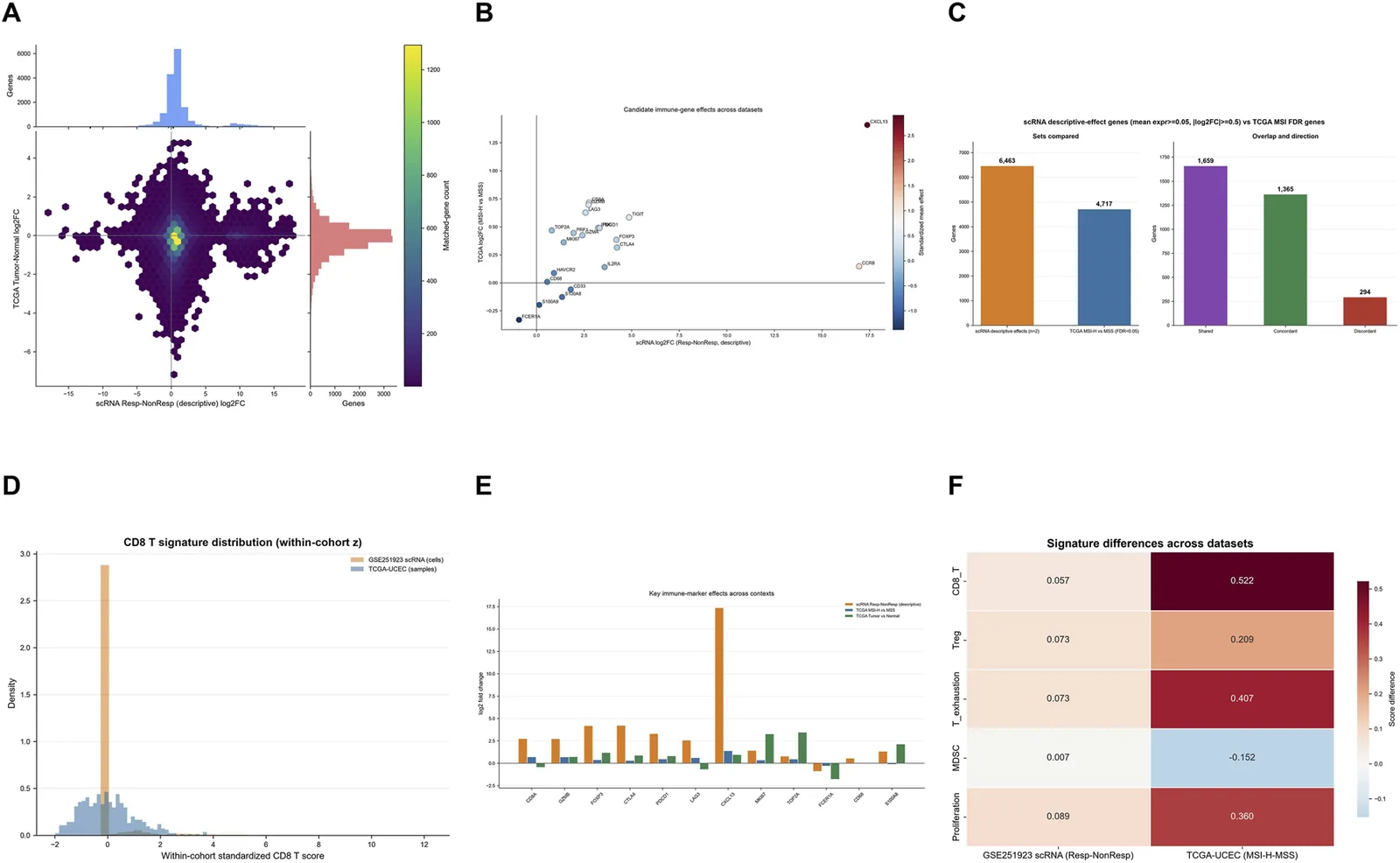
Cross-dataset contextualization of GSE251923 and TCGA-UCEC. **(A)** Hexbin density plot comparing descriptive scRNA-seq responder-versus-non-responder log_2_FC values with TCGA tumor-versus-normal log_2_FC values for 15,842 shared genes. **(B)** Directional effects of the selected immune and proliferation genes in the scRNA-seq response comparison and TCGA MSI-H-versus-MSS comparison. **(C)** Overlap between 6,463 scRNA-seq descriptive-effect genes and 4,717 TCGA MSI-H-versus-MSS FDR-significant genes. Of the 1,659 shared genes, 1,365 were directionally concordant and 294 were discordant. **(D)** Within-cohort standardized CD8 T cell score distributions in the GSE251923 single-cell and TCGA bulk tumor samples; the density magnitudes are not directly comparable because the statistical units differ. **(E)** Directional log2FC values for selected genes across the scRNA-seq response, TCGA MSI-H-versus-MSS, and TCGA tumor-versus-normal comparisons. **(F)** Signature-score differences across the scRNA-seq response and TCGA MSI contrasts. The MSI-H status is used for molecular contextualization and is not treated as a surrogate for clinical anti-PD-1 response.

The scRNA-seq descriptive-effect screening produced 6,463 genes with mean expressions ≥0.05 and |log_2_FC| ≥0.5. The broader TCGA MSI-H-versus-MSS list contained 4,717 genes with FDR < 0.05; of these, 747 genes also met the additional |log_2_FC| ≥ 0.5 threshold displayed in Figure 5F. The scRNA-seq and FDR-only TCGA sets shared 1,659 genes, including 1,365 with concordant directions and 294 with discordant directions, corresponding to 82.3% directional concordance among the overlapping genes (Figure 9C). The within-cohort standardized CD8 T cell score distributions differed markedly in terms of shape: the single-cell distribution had a sharp low-score peak and a long right tail, whereas the sample-level distribution of TCGA was broader and smoother (Figure 9D). Because the two panels represent different statistical units, i.e., individual cells and bulk patient samples, the standardized densities were used only to visualize the within-cohort distributional structure.

Across comparisons of the scRNA-seq response, TCGA MSI-H-versus-MSS, and TCGA tumor-versus-normal levels, we noted that *GZMB*, *FOXP3*, *CTLA4*, *PDCD1*, *CXCL13*, *MKI67*, and *TOP2A* generally showed positive directional effects, whereas *FCER1A* was negative in all three contexts (Figure 9E); *CD8A* and *LAG3* were positive in the response and MSI contrasts but slightly negative in the tumor-versus-normal comparison. At the signature level, responder-minus-non-responder differences in the scRNA-seq results were +0.057 for the CD8 T cells, +0.073 for the Tregs, +0.073 for T cell exhaustion, +0.007 for the MDSCs, and +0.089 for proliferation; the corresponding MSI-H-minus-MSS differences in TCGA were +0.522, +0.209, +0.407, −0.152, and +0.360, respectively (Figure 9F). Thus, CD8 T cell, Treg, T cell exhaustion, and proliferation signatures were directionally positive in both comparisons, whereas MDSC behavior was not concordant. These analyses provide cross-context prioritization of the candidate programs but do not equate MSI-H status with clinical anti-PD-1 response.

### 3.7 Exploratory *in vitro* qRT-PCR findings

Under 1% O_2_ hypoxia, both the Ishikawa and HEC-1A endometrial cancer cell lines showed higher transcript levels of *MKI67*, *FOXP3*, *CXCL13*, *PDCD1*, and *CTLA4* than under normoxia (Figure 2A). The approximate FCs in the Ishikawa cells were 3.2 for *MKI67*, 4.1 for *FOXP3*, 2.8 for *CXCL13*, 3.6 for *PDCD1*, and 2.5 for *CTLA4*; the corresponding values for the HEC-1A cells were approximately 2.9, 3.7, 3.1, 3.3, and 2.3, respectively. These findings demonstrate the hypoxia-associated inducibility of the selected transcripts in tumor cells but do not establish Treg or CD8 T cell abundance and do not model responder-versus-non-responder status directly. In the primary neutrophils from three independent donors, the endometrial-cancer ascites-conditioned medium increased *PAD4* mRNA to 3.8-fold (*p* = 0.012) and *ELANE* mRNA to 2.9-fold (*p* = 0.028) relative to the control medium (Figure 2B). These results support an ascites-associated NET-related transcriptional tendency. Because we measured only mRNA expressions, these experiments do not demonstrate functional NET formation and should be interpreted as ancillary, hypothesis-generating transcriptional evidence.

## 4 Discussion

Our integrative reanalysis showed a coordinated immune- and proliferation-related pattern across two anti-PD-1-treated endometrial cancers and TCGA-UCEC cohort. In the GSE251923 set, the responder sample contained a broader lymphoid compartment, including CD8 T cells, Tregs, B cells, plasma cells, and CD4 T cells, together with a larger proliferating cell fraction; the non-responder sample was dominated by epithelial/tumor and endothelial cells. In TCGA-UCEC, the T-cell-related signatures were higher in MSI-H than MSS tumors, while the survival analyses linked a high Treg signature to longer overall survival and a high proliferation signature to shorter overall survival. These observations support further studies of the immune composition and proliferative state as complementary features rather than isolated biomarkers.

The association between a high Treg score and longer survival may appear counterintuitive because the Tregs are commonly viewed as immunosuppressive. However, in bulk tumor data, a Treg signature can coexist with cytotoxic- and exhaustion-related T cell signals and may therefore reflect a generally immune-infiltrated microenvironment. The strong correlations among CD8 T cell, Treg, and T cell exhaustion scores in TCGA-UCEC are consistent with this interpretation. The one responder in GSE251923 also showed simultaneous enrichment of Treg, cytotoxic, and proliferating-cell compartments. These findings do not establish a protective role for the Tregs; instead, they indicate that the prognostic meaning of a Treg score depends on the surrounding immune context and molecular background (Papalexi and Satija, 2018; Wu et al., 2021; Izar et al., 2020).

Although the single-cell composition differences were pronounced, they must be interpreted at the level of two individual tumors. The large number of cells does not compensate for the absence of patient-level replication, and the separation seen on UMAP may reflect response status, patient-specific biology, sampling, or technical factors. The scarcity of Tregs and CD8 T cells in the non-responder sample further limits direct status comparisons. Thus, the most defensible use of these data is to prioritize candidate cell populations and pathways for validation rather than estimate the response-associated effect sizes in a population. The Visium results add tissue-section-level context but not true anatomical localization. The marker patterns supported broad epithelial/tumor-like, stromal/vascular, myeloid-associated, B/plasma-cell-associated, and proliferation-enriched expression compartments. Because the original tissue position files were unavailable, the displayed projections represent the expression space rather than coordinates on the tissue section. In addition, each Visium spot may contain multiple cells; thus, the observed section-level differences in the Treg, proliferation, epithelial, and ECM scores should be viewed as descriptive properties of the two sections and not as evidence of spatial colocalization or exclusion (Ståhl et al., 2016; Moses and Pachter, 2022; Moncada et al., 2020).

Cross-dataset comparisons showed that the selected cytotoxic, Treg/checkpoint, and proliferation-related genes changed in the same direction in the GSE251923 response contrast and TCGA MSI contrast. While this directional overlap is useful for candidate prioritization, the two comparisons address different questions: one contrasts two treated patients, whereas the other contrasts molecular subgroups in TCGA-UCEC. Hence, MSI-H cannot be used as a surrogate outcome for anti-PD-1 response, and differences in the statistical unit and expression scale preclude direct comparisons of the FC magnitudes.

The qRT-PCR findings also warrant a limited interpretation. The increased *MKI67*, *FOXP3*, *CXCL13*, *PDCD1*, and *CTLA4* transcripts under hypoxia show that these genes can be induced in endometrial cancer cells under stress; however, a tumor cell model cannot validate Treg abundance, T cell exhaustion, or clinical response to PD-1 blockade. Similarly, the increased *PAD4* and *ELANE* expressions after ascites-conditioned exposure suggest NET-related neutrophil transcriptional responses; these do not demonstrate NET formation or establish a causal link with immunotherapy, which would require functional assays and appropriately controlled immune models (Xiao et al., 2021; Teijeira et al., 2020; Hedrick and Malanchi, 2022).

Given these findings, several limitations of this study should be considered. First, the scRNA-seq and Visium analyses included one responder and one non-responder, so all response-related findings are exploratory. Second, the TCGA survival models were univariable and used median-split signature groups, meaning that residual confounding and information loss are possible. Third, the Visium analysis lacked tissue position files and formal cell-type deconvolutions. Fourth, the *in vitro* assays measured transcript abundances rather than immune cell functions or NET formation. Finally, the cross-dataset concordance was assessed across different biological contrasts and should not be interpreted as external validation. Future works should therefore evaluate these candidate programs in multicenter cohorts with treatment annotations, adequate numbers of responders and non-responders, and paired pretreatment and on-treatment samples. Tissue-registered spatial analysis, orthogonal immune phenotyping, and functional neutrophil assays would also help distinguish cell abundance from cell status and clarify whether the identified patterns are predictive, prognostic, or simply associated with a molecular subtype. Clinically accessible assays are also needed before the transcriptomic signatures can be used for patient selection alongside established molecular markers and the safety considerations accompanying ICI therapy (Eskander et al., 2023; Makker et al., 2022; Lyon et al., 2022).

## 5 Conclusion

Integrated analysis using data from the GSE251923 set and TCGA-UCEC enabled us to identify Treg- and proliferation-related patterns that differed between two anti-PD-1-treated endometrial cancers and were directionally supported in broader molecular contexts. In TCGA-UCEC, a high Treg signature was associated with longer overall survival, whereas a high proliferation signature was associated with shorter survival. The accompanying qRT-PCR assays showed condition-associated changes in selected transcripts but did not validate immune cell states, clinical response mechanisms, or functional NET formation. These findings provide a focused set of hypotheses for testing in independent and treatment-annotated cohorts.

## Data Availability

TCGA-UCEC gene-expression and clinical data are available from the GDC Data Portal: https://portal.gdc.cancer.gov/projects/ TCGA-UCEC. The GSE251923 data are available from the GEO database: https://www.ncbi.nlm.nih.gov/geo/query/acc.cgi?acc= GSE251923(GSM7990051–GSM7990054). The original qRT-PCR data and analysis code are available from the corresponding author upon reasonable request.
